# The Dynamic Distribution of Small-Tail Han Sheep Microbiota across Different Intestinal Segments

**DOI:** 10.3389/fmicb.2018.00032

**Published:** 2018-01-31

**Authors:** Hao Zhang, Mingxu Shao, He Huang, Shujuan Wang, Lili Ma, Huining Wang, Liping Hu, Kai Wei, Ruiliang Zhu

**Affiliations:** ^1^Shandong Provincial Key Laboratory of Animal Biotechnology and Disease Control and Prevention, Shandong Agricultural University, Tai’an, China; ^2^Shandong Provincial Engineering Technology Research Center of Animal Disease Control and Prevention, Shandong Agricultural University, Tai’an, China; ^3^Lianyungang Entry-Exit Inspection and Quarantine Bureau, Lianyungang, China; ^4^Shandong New Hope Liuhe Co., Ltd., New Hope Group, Qingdao, China; ^5^Animal Disease Prevention and Control Center of Shandong Province, Animal Husbandry and Veterinary Bureau of Shandong Province, Jinan, China

**Keywords:** gut microbiota, small-tail Han sheep, high-throughput sequencing, dynamic distribution, volatile fatty acids

## Abstract

The sheep intestinal tract is characterized by a diverse microbial ecosystem that is vital for the host to digest diet material. The importance of gut microbiota (GM) of animals has also been widely acknowledged because of its pivotal roles in the health and well-being of animals. However, there are no relevant studies on GM of small-tail Han sheep, a superior mutton variety domestic in China. In this study, the structure and distribution of gut microflora were studied by high-throughput sequencing technology. Results showed a significant difference between jejunum and cecum, jejunum, and rectum. Meanwhile, the cecum and rectum not only display higher species richness but also exhibit higher similarity of the bacterial diversity than that of the jejunum based on the results of abundance-based coverage estimator (ACE), Chao1, and Shannon indexes. *Firmicutes* and *Bacteroidetes* were the predominant phyla in cecum and rectum, while higher relative abundances of *Firmicutes* and *Cyanobacteria* were observed in jejunum. At the genus level, *Bacteroidetes*, *Ruminococcus*, *Lactobacillus*, *Flavonifractor*, and *Clostridium* were the dominant genera in the cecum and rectum. An obvious dynamic distribution of *Lactobacillus* is continuously decreasing from the jejunum to the cecum, then to the rectum, whereas the result of *Bacteroides* is completely inverse. In addition, this study also found many kinds of bacteria associated with the production of volatile fatty acids (VFA) colonized in the large intestine. This study is the first to investigate the distribution of intestinal flora in small-tail Han sheep. The findings provide an important indication for diagnosis and treatment of intestinal diseases in small-tail Han sheep, as well as offer a direction for the development of intestinal microecological preparations.

## Introduction

The gut microbiota (GM) is a diverse and complex community of microorganisms referred to as the “forgotten organ.” Studies indicate that the gut microbiome is a signaling hub that integrates environmental inputs, such as diet, with genetic and immune signals affecting the host metabolism, immunity, and response to infection (Thaiss et al., 2016). For instance, the mammalian distal intestine is a bioreactor containing anaerobic bacteria that are capable of degrading a variety of indigestible polysaccharides (Bäckhed et al., 2005). The GM is also known to provide other beneficial functions for the host, including the recycling of bile salts, production of vitamin K, and production of exogenous alkaline phosphatase (Yolton and Savage, 1976; Gilliland and Speck, 1977; Ramotar et al., 1984). The gastrointestinal tract harbors a complex population of microbes that play a fundamental role in the development of the immune system and animal health (Gillor et al., 2008; Schuijt et al., 2013). Moreover, the intestinal microbial species restrain pathogens by producing antimicrobials (such as bacteriocins and some metabolites), competing for luminal nutrients and attachment sites, as well as producing signaling molecules, which can modulate gene expression of other bacteria (Sturme et al., 2002). Recent studies have shown that the study of GM has become one of the most popular topics in the 21st century. Considering the multifaceted effects of GM, understanding animal GM for maintaining their health is important.

The GM exhibits a large quantity and a complex composition known as the second genome of the body. In the past, traditional culture-dependent methods would be indispensable to understand the types of the GM. However, more than 50% of GM cannot be cultured out of the gut. Thus, scientists cannot make a description particularly about GM. To date, high-throughput DNA sequencing technology offers a convenient and fast tool in describing the secret between the animal body and the GM (Shendure and Lieberman, 2012; Marx, 2013). For instance, the study by Round and Mazmanian (2009) demonstrated that developmental aspects of the adaptive immune system are influenced by bacterial colonization of the gut. Some studies proved that the gut communities are influenced not only by geographically and culturally distinct settings but also by age (Dominguez-Bello et al., 2011; Yatsunenko et al., 2012) with the assistance of high-throughput sequencing technology. Moreover, in recent years, gut microbes and the relationship between children’s growth and development is gradually becoming a frontier research field. Blanton et al. (2016) have shown that *Lactobacillus plantarum* strains in the intestinal flora can maintain growth hormone activity through the signaling pathway of the liver, which can overcome the growth hormone resistance. Dominguez-Bello et al. (2016) demonstrated that “microbial bath” can repair the intestinal flora of cesarean section newborn. Undoubtedly, these studies, which are good for the development of clinical medicine, are all inseparable from high-throughput sequencing technology.

Small-tail Han sheep, a local variety, is widely raised in China because of its performance advantages, such as fattening ability, low fat rate, strong disease resistance, and genetic performance stability. The preservation of these excellent properties is not only connected with its own genetic genes but also probably with intestinal microorganisms. However, to date, our knowledge about the GM of small-tail Han sheep is minimal. In the present study, we use high-throughput sequencing based on Illumina MiSeq platform to analyze the microbial community in the jejunum, cecum, and rectum of the small-tail Han sheep. The results present the distribution and difference of the different populations of microorganisms in these three intestinal segments, as well as the dynamic distribution of the related bacteria in the entire intestinal tract.

## Materials and Methods

### Ethics Statement

The animal procedures were approved by the Animal Care and Use Committee of Shandong Agricultural University (Permit number: 20010510) and performed in accordance with the “Guidelines for Experimental Animals” of the Ministry of Science and Technology (Beijing, China). This study did not involve any endangered or protected species.

### Animals and Sample Collection

Three male 12-month-old healthy small-tail Han sheep were obtained from a commercial feedlot (Shandong Province, China). The sheep’s diets were composed of 58.21% green hay, 16.77% corn, 12.53% alfalfa, 8.24% soybean meal, wheat bran 3.13%, 0.11% CaHPO4, 0.90% NaCl, and 0.11% premix. The animals were fed under the standard livestock management practices. Then, the sheep were euthanized by intravenous injection of euthanasia solution, and the fresh samples (10 g) of different intestinal segments, including jejunum (J1, J2, and J3), cecum (C1, C2, and C3), and rectum (R1, R2, and R3) were collected simultaneously. The samples were transported to laboratory within 2 h in ice and stored at -80°C.

### DNA Extraction, PCR Amplification and Illumina MiSeq Sequencing

Genomic DNA was extracted from each sample by using TIANamp Genomic DNA Kit (TIANGEN Bio-Tek Co., Ltd., Beijing, China) following the manufacturer’s instructions, and the extracts were purified by using DNA Purification Kit (Tiangen DNA gel extraction kit, China). Subsequently, generation sequencing library preparations and Illumina MiSeq sequencing were conducted at GENEWIZ, Inc. (Beijing, China). The quality of the DNA was checked by 1% (w/v) agarose gel electrophoresis and the concentration of DNA was measured with a UV–Vis spectrophotometer (Nano-Drop 2000, United States). 5–50 ng DNA was used to generate amplicons using a MetaVx^TM^ Library Preparation kit (GENEWIZ Inc., South Plainfield, NJ, United States). The different hypervariable regions of the 16S rRNA (V3, V4, and V5) were amplified using the special primers (V3 and V4 regions for forward primers containing the sequence 5′-CCTACGGRRBGCASCAGKVRVGAAT-3′ and reverse primers 5′-GGACTACNVGGGTWTCTAATCC-3′, V4 and V5 regions for forward primers containing the sequence 5′-GTGYCAGCMGCCGCGGTAA-3′ and reverse primers 5′-CTTGTGCGGKCCCCCGYCAATTC-3′). In addition to the 16S target-specific sequence, the adaptor sequences allowing uniform amplification of the library with high complexity ready for downstream next-generation sequencing on Illumina Miseq were contained as well. DNA libraries were validated by Agilent 2100 Bioanalyzer (Agilent Technologies, Palo Alto, CA, United States) and quantified by Qubit and real-time PCR (Applied Biosystems, Carlsbad, CA, United States). Then, DNA libraries were multiplexed and loaded on Illumina MiSeq instrument following the manufacturer’s instructions (Illumina, San Diego, CA, United States). Sequencing was performed using a 2 × 250 or 2 × 300 paired-end configuration. Image analysis and base calling were conducted by the MiSeq Control Software on the MiSeq instrument. The sequences of V3, V4, and V5 were processed, spliced, and analyzed by GENEWIZ (Beijing, China). Taxonomy analysis was carried out on QIIME^1^. Raw sequence data of this study have been deposited to the NCBI Sequence Read Archive with accession no. SRP127379.

### Bioinformatics and Statistical Analysis

Sample reads were assembled by using PANDAseq (v2.7), Trimmomatic (v0.30), and Usearch (v8.0). Sequences shorter than 400 bp or containing homopolymers and ambiguous bases were removed, and adapter/index sequences were trimmed. High-quality sequences were binned into operational taxonomic units (OTUs) using UCLUST (Edgar, 2010) with 97% sequence identity threshold. All cleaned sequences were classified into taxa using the Greengenes 16SrRNA Gene Database (Desantis et al., 2006). The representative sequences were taxonomically classified by using the ribosomal database project (RDP) classifier. The relative abundances of the phylum, family, and genus levels were plotted as bar graph, and the relative abundances of the genus levels were showed as heatmap. The numbers of the share genera were showed as the Venn diagram. Linear discriminant analysis Effect Size (LEfSe) was used to analyze the differences of microbiome between groups. Alpha diversity analysis included Shannon index, Chao1. Beta diversity included both unweighted and weighted Unifrac distances calculated for 10 times of subsampling. These distances were visualized by principal coordinate analysis (PCoA). The criterion of significance was conducted at *P* < 0.05. Data were expressed as mean ± standard deviation (SD) and were performed using SPSS 17.0.

## Results

### Microbial Diversity Index Analysis of Different Intestine Segments

The contents of the jejunum, cecum, and rectum were collected to conduct high-throughput Illumina MiSeq sequencing to delineate the bacterial community composition among different intestinal segments of small-tail Han sheep. Targeting the V3, V4, and V5 hypervariable regions, 1,001,703 sequences were produced for nine samples after chimera checking and filtering out. Each sample has 111,300 sequences on average approximately. OTUs were defined as a read sharing 97% nucleotide-sequence identity.

For alpha diversity measurements, the bacterial diversity and richness were assessed by using the Shannon index, Chao1, abundance-based coverage estimator (ACE) and Good’s coverage. Besides, the Good’s coverage of each sample was over 97%, indicating that the 16SrDNA sequences identified in these samples represent the majority of bacteria present in the samples of this study (**Table 1**). The highest microbial richness of samples were found in the cecum and rectum, the average of Chao1 index varied from 6211.5100 to 6369.5200, and the average of ACE index varied from 6510.6867 to 6692.9433. The richness of jejunum sample was lower relatively than those of cecum and rectum, and the average of Chao1 and ACE indexes were 2227.0000 and 2349.0733, respectively. Similarly, the cecum and rectum samples had the highest microbial diversity, the average of Shannon index were between 8.8627 and 8.9757, while the average Shannon index of jejunum was about 5.9060 (**Table 1**). The results showed no significant differences in Shannon diversity between the cecum and rectum samples. Meanwhile, the cecum and rectum samples displayed significant difference with the jejunum samples. For community richness comparison, both the abundance-based coverage estimator (ACE) and Chao1 showed that the cecum and rectum exhibited significantly higher number of observed and estimated OTUs than the jejunum. No significant differences in richness were observed between the cecum and rectum. This result demonstrated that bacterial diversity and abundances of the cecum and rectum are greater than that of jejunum (**Table 1**).

**Table 1:** Collation results of alpha diversity analysis.

Sample	ACE	Chao1	Shannon	Good’s coverage
Jejunum	2349.0733 ± 1.0517^a^	2227.00 ± 977.8583^a^	5.9060 ± 1.7832^a^	0.9957 ± 0.0012
Caecum	6692.9433 ± 2.0356^b^	6369.5200 ± 1.7785^b^	8.8627 ± 0.5653^b^	0.9840 ± 0.0089
Rectum	6510.6867 ± 1.0516^b^	6211.5100 ± 812.9195^b^	8.9757 ± 0.5539^b^	0.9882 ± 0.0084


*Data are expressed as average ± standard deviation (*n* = 3). ^a,b,c^Within a column, means for the individual subsamples having different superscripts differ significantly (*P* < 0.05).*

### Beta-diversity Analysis of the Microbial Community of Different Intestine Segments

**FIGURE 1:**
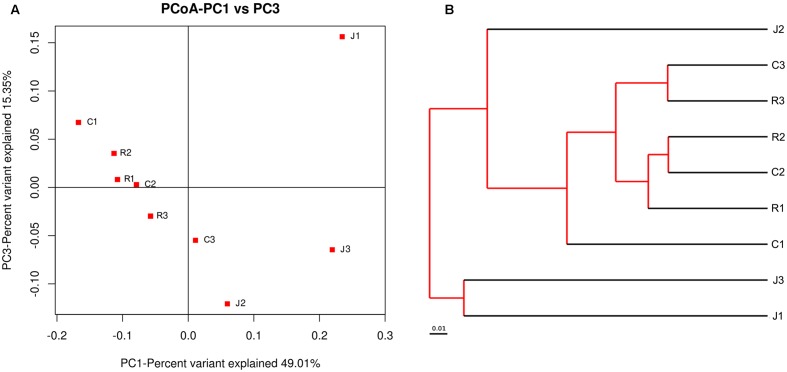
Differences in bacterial community structures and relationship between all of the samples. **(A)** Principal Coordinate Analysis (PCoA) of bacterial community structures of the gut microbiota of the three sample groups. Each symbol represents each gut microbiota. PCoA shows distinct bacterial communities between different samples. **(B)** The UPGMA tree analysis of samples in evolution.

The relationships between the community structures of the small-tail Han sheep GM were examined by using the PCoA. The results showed that the microbiota of the jejunum samples were distinct from those of the samples of the cecum and rectum. No significant differences in community structure were observed between the samples of the cecum and rectum (**Figure 1A**). The relationships between the community structures revealed by PCoA were further tested by comparing the between-group weighted Unifrac distances and unweighted pair-group method with arithmetic mean (UPGMA) tree. Consistent with the PCoA plot, the between-group distances of the cecum and jejunum, rectum and jejunum were significantly higher than that of the cecum and rectum. The UPGMA tree showed that the cecum and rectum of the small-tail sheep have high similarity in evolution (**Figure 1B**). These data suggested that the microbial community structures between the jejunum and cecum, jejunum and rectum were significantly different, whereas those between the cecum and rectum were not significantly different.

Interestingly, we draw a similar conclusion under alpha-diversity analysis and beta-diversity analysis that the cecum and rectum display high similarity in microbial diversity and community structure, whereas the differences between the cecum and rectum were not significant. Meanwhile, both of them display significant difference compared with the jejunum.

### Bacterial Community Composition at Different Taxonomical Levels

**FIGURE 2:**
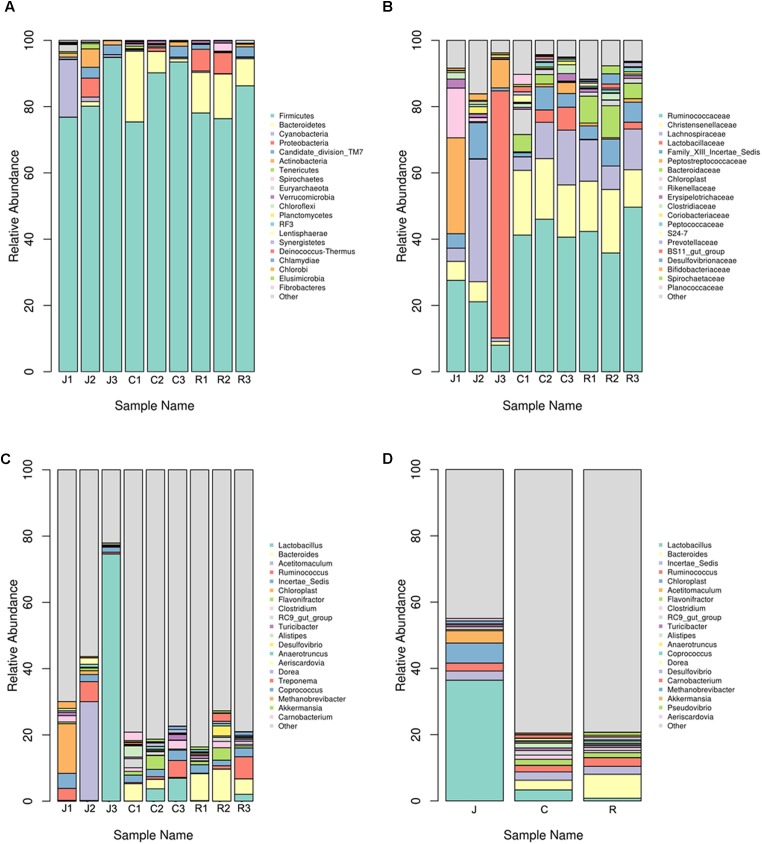
Microbial composition of different samples. Each bar represents the average relative abundance of each bacterial taxon within a group. The top 21 abundant taxa are shown. **(A)** Taxa assignments at Phylum level. **(B)** Taxa assignments at Family level. **(C)** Taxa assignments at Genus level. **(D)** Between-group taxa assignments at Genus level.

In the following work, we analyzed the gut bacterial community composition and structure in different taxonomical levels. According to the phylum assignment result, *Firmicutes* were the predominant phylum in the nine samples. *Bacteroidetes* were the secondary phylum in the cecum and rectum, whereas in the jejunum, the secondary phylum was *Cyanobacteria.* The high abundance of phylum *Proteobacteria* was found in J2, R1, and R2 samples (**Figure 2A**). Besides the phylum, bacterial abundance was also analyzed specifically at other taxonomic units, family (**Figure 2B**), and genus (**Figure 2C**).

On the family level, no significant differences were observed between the six samples of the cecum and rectum. The top five predominant populations in the cecum and rectum were *Ruminococcaceae*, *Christensenellaceae*, *Lachnospiraceae*, *Bacteroidaceae*, and *Lactobacillaceae*. However, the three samples of the jejunum were significantly different. The top five predominant populations in the J1 sample were *Ruminococcaceae*, *Peptostreptococcaceae*, *Lachnospiraceae*, *Christensenellaceae*, and *Clostridiaceae*. In the J2 sample, the top five predominant populations were *Lachnospiraceae*, *Ruminococcaceae*, *Bifidobacteriaceae*, *Christensenellaceae*, and *Coriobacteriaceae*. Meanwhile, the most abundant families in the J3 sample were *Lactobacillaceae*, *Ruminococcaceae*, and *Peptostreptococcaceae*, which almost made up the total bacterial community.

**FIGURE 3:**
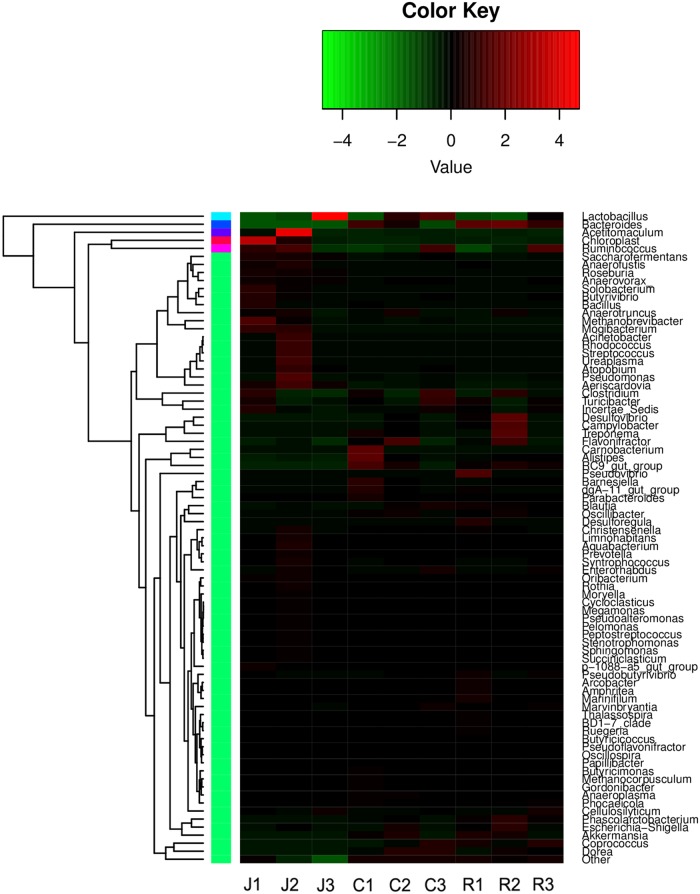
Heatmap of hierarchy cluster results for the abundance of genus in different intestine segments.

At the genus level, no significant differences were observed between the six samples from the cecum and rectum. The top five predominant populations in the cecum and rectum were *Bacteroides*, *Ruminococcus*, *Lactobacillus*, *Clostridium*, and *Flavonifractor* (**Figures 2C,D**, **3**). However, similar to the family level, the three samples from the jejunum were significantly different. In the J1 sample, the top three predominant populations were *Ruminococcus, Clostridium* and the archaea *Methanobrevibacter*. The top three predominant populations in the J2 sample were *Acetitomaculum*, *Ruminococcus* and *Aeriscardovia*. However, *Lactobacillus* was the most abundant genus in the J3 sample (**Figures 2C**, **3**). Besides, in the jejunum samples, the top five predominant populations were *Lactobacillus*, *Acetitomaculum*, *Ruminococcus*, *Clostridium*, and *Methanobrevibacter* (**Figure 2D**). The most important factor is that the result found the obvious laws of change between *Bacteroides* and *Lactobacillaceae. Lactobacillus* proportion gradually decreases from the jejunum to the cecum, then to the rectum, whereas the result of *Bacteroides* is completely inverse (**Figure 2D**).

**FIGURE 4:**
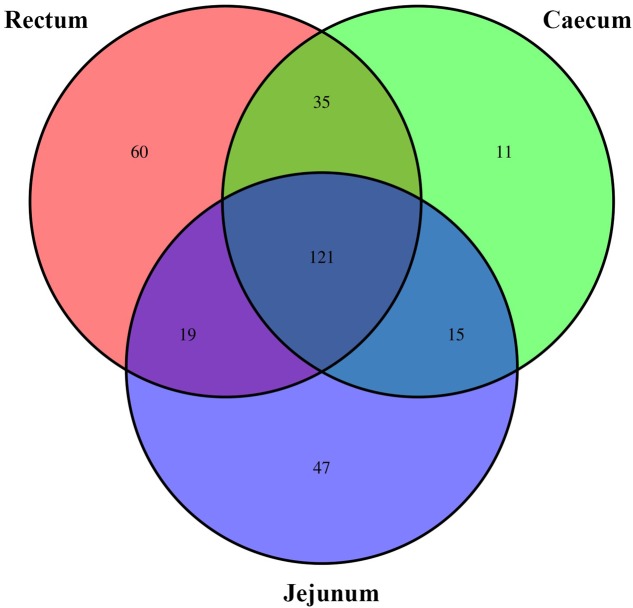
The scalar-Venn representation of share genera among microbiota in different groups.

On the genus level, the Venn diagram (**Figure 4**) reflects the difference between the small-tail Han sheep microbial community. The total OTUs in all groups are 308. Up to 202, 182, and 235 OTUs belong to the jejunum, cecum, and rectum, respectively. Besides, 35, 19, and 15 OTUs belong to the cecum and rectum, jejunum and rectum, and jejunum and cecum, respectively. This finding indicated that the cecum and rectum are similar in gut bacterial community quantity.

### Differences in Bacterial Communities between the Jejunum, Cecum, and Rectum

**FIGURE 5:**
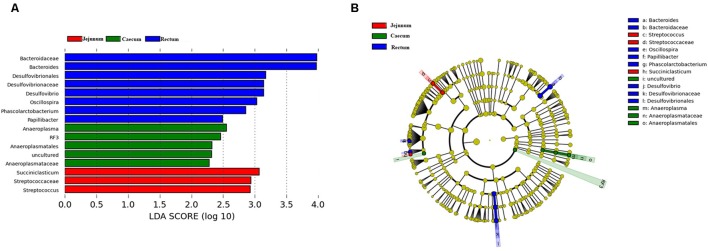
Bacterial taxa significantly differentiated between the jejunum, caecum, and rectum samples identified by linear discriminant analysis effect size (LEfSe) using the default parameters. **(A)** Histogram of the LDA scores computed for bacterial taxa differentially abundant among different groups. **(B)** Bacterial taxa that were differentially abundant in different groups visualized using a cladogram.

We performed LEfSe on 16 top taxa (average relative abundance >0.0001) to identify the bacterial taxa that were significantly differentiated between groups. **Figure 5** shows bacterial taxa differentially represented between the jejunum, cecum, and rectum. Eight bacterial taxa were significantly abundant in the rectum (e.g., *Bacteroides*, *Desulfovibrio*, *Oscillospira*, *Phascolarctobacterium*, and *Papillibacter*) and five bacterial taxa were significantly abundant in the cecum (e.g., *Anaeroplasma*, *RF3*). Meanwhile, only three taxa were overrepresented in the jejunum (e.g., *Succiniclasticum* and *Streptococcus*). In both species and quantity, the flora in the rectal samples was significantly higher than that in the jejunum, followed by the cecum. Besides that, *Bacteroides* are one of 16 top taxa abundant in the rectum. This finding is also consistent with the changes of *Bacteroides* mentioned above.

## Discussion

Gut microbiota is known as the “second genome” playing a significant part in the animal body. Studies have shown that intestinal microbes can affect body weight and digestive capacity and resist the risk of infection and autoimmune diseases. To date, the GM of some animals, including sheep, cow, and other ruminants, had been studied. Wang et al. (2017) has investigated the bacterial composition of sheep gastrointestinal microbiota, and showed that *Ruminococcus flavefaciens*, *Butyrivibrio Fibrisolvens*, and *Selenomonas ruminantium* were three most dominant species in sheep gastrointestinal tract. Zeng et al. (2015) investigated the composition and quantification of GM in Chinese Mongolian sheep and first revealed the cellulolytic bacterial community in these sheep. In this study, we analyzed the bacterial diversity and abundance in distinct small-tail Han sheep intestinal samples, and the results showed that the cecum and rectum harbored the higher richness and diversity of bacteria compared with the jejunum. It is more meaningful for us to first find some specific bacteria in rectum.

At the phylum level, we found that the structure of bacterial community in the intestinal tract was similar to Chinese Mongolian sheep (Zeng et al., 2015). *Firmicutes* and *Bacteroidetes* were the predominant phylum in cecum and rectum, while higher relative abundances of *Firmicutes* and *Cyanobacteria* were observed in jejunum of small-tail Han sheep. For the ruminants, Firmicutes plays an important role on degrading the fiber and cellulose (Thoetkiattikul et al., 2013). *Bacteroidetes* can promote digestion and increase utilization of complex carbohydrates (Spence et al., 2006). *Cyanobacteria* has many special functions including obligate anaerobic fermentation, syntrophic H2-production, nitrogen fixation, and synthesis of vitamin B and K21 (Rienzi et al., 2013). Besides that, the high abundance of phylum *Proteobacteria* was found in some large intestine (jejunum and rectum) samples. Previous studies had reported that high abundance of phylum *Proteobacteria* was found in the small intestine of sheep and cattle (Looft et al., 2014; Mao et al., 2015; Wang et al., 2017). Thus the colonization of phylum *Proteobacteria* may be variable in different animal individuals.

Furthermore, the GM of small-tail Han sheep referring to their correlation with different intestinal tracts was characterized at the genus level. We found several bacterial taxas, such as *Lactobacillus* and *Ruminococcus* were overrepresented in the jejunum, cecum, and rectum. This point is in agreement with the previous studies, in which they also reported that *Lactobacillus* and *Ruminococcus* were dominant in the small and large intestines (Lu et al., 2003; Wang et al., 2017). *Lactobacillus*, wherein members of which are well-known lactate-producing probiotics, are wildly used to improve animal digestion efficiency. *Ruminococcus* plays an important role in dissolving nutrients, such as cellulose and hemicellulose (Wood et al., 1982; Doerner and White, 1990). *Ruminococcus* can produce cellulases and hemicelluloses (Suen et al., 2011), which were important for the primary consumer. The number of *Ruminococcus* overrepresented in the jejunum may indicate that carbohydrate metabolism is active in the metenteron of small-tail Han sheep. In addition, some *Ruminococcus* are involved in biological hydrogenation of unsaturated fatty acid (Huws et al., 2011) and degrade aromatic compounds, cinnamic acid, and crotonic acid ester (Defnoun et al., 2003).

Although similar microbial richness was observed between the jejunum, cecum, and rectum, the community structures are significantly different between different intestinal tracts. In our results, *Bacteroides* and *Flavonifractor* belonged to the cecum and rectum samples peculiarly, however, the microbiota in jejunum was strikingly different from those in the cecum and rectum samples. For instance, *Clostridia* occupied a relatively large proportion in the jejunum and cecum sample, while the *Acetitomaculum* was overrepresented in the jejunum.

As intestinal superior bacteria, *Bacteroides* can break down polysaccharides and improve nutrient utilization rate (Bäckhed et al., 2004), speed up the formation of intestinal mucosa (Stappenbeck et al., 2002), and develop the immune system to improve the host’s immune system (Hooper, 2004). *Bacteroides* can also maintain the intestinal microecological balance (Sears, 2005). *Flavonifractor* exhibits the ability to degrade flavonoids, serving as substrates for the human GM and can be transformed by various bacterial species (Blaut et al., 2003). No enzyme exists in the intestine to decompose the flavonoids in the food into flavonoid ligands. Only the microorganisms in the colon can hydrolyze the β-glycosidic linkages and release the free flavonoid ligands (Hollman et al., 1997). This conclusion is in line with our results that *Flavonifractor* were overrepresented in the samples from the jejunum, cecum, and rectum. The large intestine as an important place for secondary digestion of food plays an important role in improving food utilization. Thus, we predicted that the dominant bacteria (e.g., *Bacteroides*, *Lactobacillus*, *Ruminococcus*, and *Flavonifractor*) not only can maintain the healthy and stable level of the intestinal tract, but also importantly take part in the digestion and absorption of residual nutrients and prevent nutrients from running away largely.

*Acetitomaculum* belonging to acetogenic bacteria can utilize formate, glucose, and CO and participate in hydrogen utilization in the rumen ecosystem (Greening and Leedle, 1989). *Acetitomaculum* is an inhabitant of the rumen (Mao et al., 2015). We predicted that huge number of *Acetitomaculum* in the jejunum may come from the rumen with chyme but also possibly participates in nutrient digestion in the small intestine. *Clostridia* can influence the host animal positively or negatively. Among which, *Clostridium tetani*, *C. botulinum*, and *C. difficile* generally make a negative influences on animal health (Songer, 2006; Attwood et al., 2006). Conversely, some kinds of *Clostridium* may be beneficial for improving digestion of complex organic matter (Leser et al., 2002; Ozutsumi et al., 2005). Thus it is necessary to investigate the species of *Clostridia* in jejunum and cecum of small-tail Han sheep, which may be related to their specific biological characteristics.

*Succiniclasticum* and *Streptococcus* are specific bacteria in the jejunum samples. Study has shown that *Succiniclasticum* specializes in fermenting succinate and converting it to propionate (Gylswyk, 1994). As an important glucose precursor of ruminants, propionic acid fermentation can provide great amount of energy for the body. The small intestine is the main place for the absorption of nutrients, thus, large number of *Succiniclasticum* in the jejunum is normal. For sheep or goat, almost all studies about *Succiniclasticum* focused on rumen, while our findings imply propionic acid fermentation based on Succiniclasticum continuously proceed in the small intestine. As a resident colony of the intestine, *Streptococcus* had been proved as the main group of bacteria in the jejunum (Guarner, 2006), which is consistent with our results.

In the cecum samples, *Anaeroplasma* is dominant and has been examined for enzymic activities of aromatic amino acid and carbohydrate metabolism (Petzel and Hartman, 1990). The cecal contents contain high levels of ammonia, isobutyric acid, and isohexanoic acid because of protein hydrolysis and deaminase activity. *Anaeroplasma* may be involved in this metabolic process and may exhibit the ability to decompose nutrients to produce isobutyric acid and isovaleric acid.

*Bacteroides*, *Desulfovibrio*, *Oscillospira*, *Phascolarctobac-terium*, and *Papillibacter* are peculiar in the rectum samples. Besides the description referred above, studies have also found that the obesity is correlated with a shift in the abundance of *Bacteroidetes* (Callaway et al., 2010; Bo et al., 2015; Min et al., 2015). In general, lean meat rate of small-tail Han sheep is relatively higher than other sheep. Therefore, we predict that *Bacteroides* may contribute to the low fat rate of small-tail Han sheep. To our knowledge, *Desulfovibrio*, *Oscillospira*, *Phascolarctobacterium*, and *Papillibacter* were found in rectum of small-tail Han sheep for the first time. A recent study has reported the high abundance of genus *Desulfovibrio* and *Oscillospira* was found in rumen of cattle or sheep (Mackie et al., 2003; Gulino et al., 2013). The product decomposed by *Desulfovibrio* and *Oscillospira* (e.g., butyrate, Hydrogen sulfide etc.) can protect the gastrointestinal tract and promote food digestion (Fournier et al., 2003; Mackie et al., 2003; Maekawa et al., 2005). *Phascolarctobacterium* and *Papillibacter* are also proved to be related to volatile fatty acids (VFA) production, such as acetate and butyrate (Dot et al., 1993; Watanabe et al., 2012). Faichney (1969) reported the VFA content in sheep large intestine was about 8% of total VFA production, thus these bacteria in rectum of small-tail Han sheep may be important participant in maintaining VFA stability.

## Conclusion

This study characterized the GM in different intestinal segments of small-tail Han sheep. Results showed a significant difference between jejunum and other two large intestines (cecum and rectum). The cecum and rectum had a similar bacterial community, but showed a higher bacterial richness than jejunum. An obvious dynamic distribution of *Lactobacillus* was observed, which it was continuously decreasing along jejunum to rectum, whereas the distribution of *Bacteroides* was completely inverse. In addition, we also found that the large intestine distributes many kinds of bacteria associated with the VFA production. These findings serve as immediate targets for further studies on investigating their roles on the growth of small-tail Han sheep, and they also can be considered as normal instruction for diagnosis and detection of intestinal diseases. Moreover, this study also provides theoretical foundation for filtrating probiotics and developing intestinal microecological preparations.

## Author Contributions

RZ, KW, LH, HZ, and MS designed the research. HZ, MS, HH, SW, LM, and HW performed the research. HZ, MS, KW, and RZ analyzed the data. HZ, MS, LH, KW, and RZ wrote the paper.

## Conflict of Interest Statement

The authors declare that the research was conducted in the absence of any commercial or financial relationships that could be construed as a potential conflict of interest.

## Footnotes

1 http://qiime.org/tutorials
